# Blood Tracer Kinetics in the Arterial Tree

**DOI:** 10.1371/journal.pone.0109230

**Published:** 2014-10-09

**Authors:** Elias Kellner, Peter Gall, Matthias Günther, Marco Reisert, Irina Mader, Roman Fleysher, Valerij G. Kiselev

**Affiliations:** 1 Department of Radiology, Medical Physics, University Medical Center Freiburg, Freiburg, Germany; 2 Siemens AG, Healthcare Sector, Erlangen, Germany; 3 Fraunhofer MEVIS, Institute for Medical Image Computing, Bremen, Germany; 4 Department of Neuroradiology, University Medical Center Freiburg, Freiburg, Germany; 5 Gruss Magnetic Resonance Research Center, Department of Radiology, Albert Einstein College of Medicine, New York, New York, United States of America; University of Arizona, United States of America

## Abstract

Evaluation of blood supply of different organs relies on labeling blood with a suitable tracer. The tracer kinetics is linear: Tracer concentration at an observation site is a linear response to an input somewhere upstream the arterial flow. The corresponding impulse response functions are currently treated empirically without incorporating the relation to the vascular morphology of an organ. In this work we address this relation for the first time. We demonstrate that the form of the response function in the entire arterial tree is reduced to that of individual vessel segments under approximation of good blood mixing at vessel bifurcations. The resulting expression simplifies significantly when the geometric scaling of the vascular tree is taken into account. This suggests a new way to access the vascular morphology in vivo using experimentally determined response functions. However, it is an ill-posed inverse problem as demonstrated by an example using measured arterial spin labeling in large brain arteries. We further analyze transport in individual vessel segments and demonstrate that experimentally accessible tracer concentration in vessel segments depends on the measurement principle. Explicit expressions for the response functions are obtained for the major middle part of the arterial tree in which the blood flow in individual vessel segments can be treated as laminar. When applied to the analysis of regional cerebral blood flow measurements for which the necessary arterial input is evaluated in the carotid arteries, present theory predicts about 20% underestimation, which is in agreement with recent experimental data.

## Introduction

Blood flow is a process of fundamental physiological importance. Today, several imaging modalities of positron emission tomography (PET), magnetic resonance imaging (MRI) and computer assisted tomography (CT) are available for its assessment using different kinds of tracers. Modeling the tracer kinetics in the vasculature is a pivotal component of these methods. The time-dependent tracer concentration in blood is described by the indicator dilution theory introduced by Stewart in his pioneering work in 1893 [1]. The theory has been validated and refined, in particular in Refs [2]–[4] and states the general linear dependence of the local tracer concentration on its input somewhere upstream. In observable terms, the tracer transport manifests itself as delay and dispersion of tracer concentration in the blood stream, which is described by the impulse response function *h(t)*. In the present context, we refer to *h(t)* as the transport function.

Determining the shape of *h(t)* is the technical focus of measurement techniques aimed at regional flow estimation [5]. Relation of this shape to the morphology of the vascular system remains however elusive. As mentioned by Zierler in 2000 [6], after enough time has been spent “in a fruitless search for a mechanism… of delay and distribution” the pioneers decided to treat the vascular system as a black box with only input and output accessible to observations.

The aim of this work is to shed light on the black box by analyzing theoretically the form of the transport function, *h(t)*, for the entire arterial system of an organ. It is known that dispersion of blood flow occurs already in individual vessel segments due to dispersion of flow velocities of blood particles. In particular, treating vessel segments as straight cylindrical tubes with laminar (Poiseuille) flow results in a simple expression for the transport function in each segment [7], [8]. However, even this simplest case is controversial; in particular, the results of two cited papers contradict each other. We resolve this contradiction by demonstrating that the observed transport function actually depends on the measurement principle.

We further analyze the entire arterial system with the topology of a tree. It is obvious that bifurcations disturb the blood flow. We show that approximating their effect by good mixing of blood simplifies the problem enormously: The transport function between two locations in the arterial tree is then expressible as a convolution of transport functions in individual vessel segments connecting these locations. Rationale and limitations of this approach are discussed in detail.

According to this result, calculation of the total transport function of the entire tree — from a stem vessel to thin branches — requires knowledge of flow patterns in all vessel segments, which is impractical. We show that the amount of required information can be drastically reduced by using scaling laws that describe the morphology of the arterial system in statistical terms. In particular, we analyze the self-similarity model [9] and Murray's law of branching [10] of the arterial tree with Poiseuille flow in its segments. In such a system, the flow velocities everywhere in the ramifying arterial tree are described by a single parameter — the flow velocity in the stem vessel — allowing explicit expression for the total transport of the tree to be obtained.

The established relation between the flow and the morphology of the arterial tree suggests probing statistical characteristics of arterial system in vivo using experimentally determined transport functions. However, it is an ill-posed inverse problem. We demonstrate this nature of the problem by an exemplary measurement using MRI for magnetic labeling of water proton spins in large brain arteries.

We apply the developed theory to MRI measurements of cerebral blood flow in which the tissue response to the injected tracer bolus should be normalized to the tracer inflow in the feeding artery. Due to technical limitations, the latter is commonly measured in a large artery, which is distal to the tissue of interest. We show that such measurements underestimate the blood flow by about 20% in agreement with recent experimental comparison between MRI and PET.

These results are presented next, followed by Discussion and Conclusion. The Methods section presents the necessary derivations and details of the experiment.

## Results

**Figure 1:**
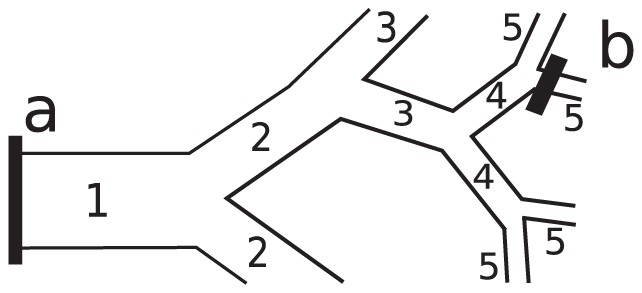
Vascular tree model: The individual segments are considered as straight cylindrical tubes with rigid walls. The bifurcations are treated symmetric on average as discussed in the text. Within this approximation, the present Weibel's generation numbers [48], are equivalent to the Horton – Strahler's order numbers [49], [50] up to the reversed order. Tracer transport from the location *a* in the stem segment to a location *b* is described by Eq. 2 with *N* = 4 in this example.

Results are formulated in terms of the transport function, *h(t)*, establishing the linear relation of the tracer concentration time course, 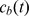, at a location *b* somewhere in the vasculature to the concentration 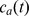 at an upstream location *a*:
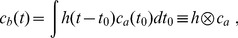
(1)where ⊗ is used as a short notation for the time convolution in what follows. Figure 1 illustrates the model of the arterial tree used in this work. The generation number *n* = 1 is assigned to the stem vessel, *n* = 2 to the daughter vessels etc. The arterial tree ends at capillaries that form a mesh network rather than a tree and are outside the scope of the present model.

The function *h(t)* can be understood as the distribution of transit times of infinitely small blood volumes between the sites *a* and *b*. As any distribution, it is normalized to unity for non-decaying tracers. Reduction of this norm accounts for tracer decay if any.

### Transport in the entire arterial tree

Central approximation of the present model is good mixing of blood at bifurcations, at least in the statistical sense in large vascular networks. Good mixing at bifurcations implies statistical independence between the transit times in two successive vessel segments. Accordingly, the two-segments transit time distribution takes the form of the time convolution of the corresponding single-vessel distributions. Applying this rule iteratively, we obtain the distribution of transit times for a system of *N* segments (Fig. 1) as a convolution chain of distributions for each segment:
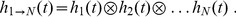
(2)where 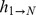 denotes the transport function from the beginning of the first to the end of the *N*th segment and *h_n_* are the individual one-segment functions.

Equation 2 is general enough to accommodate any model of the arterial tree, the only assumption here is good mixing of blood at the bifurcations. The transport functions for the individual segments depend on their dimensions and flow velocity. Such information may be available for large arteries, but the number of required characteristics rapidly becomes unavailable and intractable when analysis is extended to numerous small arteries. This suggests utilizing a statistical approach that is based on the averaged characteristics of small blood vessels. In this spirit, the arterial tree is often described by scaling laws that characterize how vessel dimensions and flow change from large to small arteries. Such relations radically reduce the number of independent parameters in the convolution chain in Eq. 2.

We now illustrate this reduction using the Murray's law [10] combined with the simplest self-similar model of the arterial tree [9]. The Murray's law derives physiological properties of the arterial system from the principle of minimal work. According to this law, the blood flow in a vessel is proportional to the cube of its radius, *r*. The self-similarity implies that all linear dimensions of vessels reduce by the same factor with increasing generation number. In particular, the length of vessel segments, *l*, scales in proportion to their radius. A scaling law for the blood velocity, 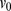, follows from this scaling and the flow conservation resulting in proportionality to the vessel radius, 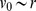, [10]. The transit time through vessel segments, 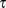, scales as 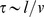, which is independent of the vessel radii and thus of vessel generation. We demonstrate below for the case of laminar flow that this time constant is the only parameter defining the shape of the transport function. Under this condition, the convolution chain in Eq. 2 simplifies to a power in the sense of convolution, 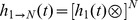. This corresponds to the algebraic power in the Fourier representation
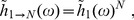
(3)where tilde denotes the Fourier transformed functions.

This result represents an enormous simplification: Tracer transport in large parts of arterial tree in which the flow is laminar is expressed via transport in a single vessel segment described below. We proceed now with a demonstration that the explicit form of the transport function depends on the measurement technique.

### Dependence on the measurement principle

**Figure 2:**
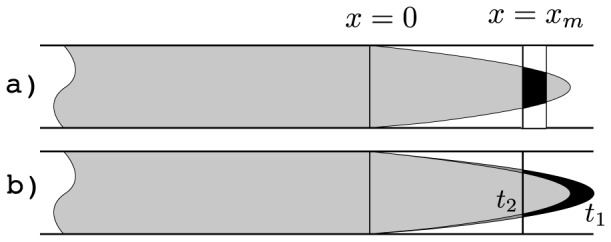
There are two different measures for the tracer concentration in a laminar flow: a) Either by determining the fraction of the labeled volume (black) in the infinitesimal measurement volume near *x* = *x_m_* at one time instant (snapshot-type) or b) by determining the amount of labeled (black) and non-labeled fluid which has passed a cross section in an infinitesimal time interval [*t*_1_,*t*_2_] and is mixed afterwards (flow-type). In the first case, all laminae contribute with equal weights, whereas in the latter they are weighted with their corresponding velocities, such that the two measurement types lead to different results.

Consider two basic ways of measuring the tracer concentration in a given vessel segment with laminar flow (Fig. 2). The first measurement class comprises snapshot-type measurements in which the tracer concentration is assessed instantly in a given volume of a vessel. Such a measurement can be performed for instance using properly collimated X-rays to sense the presence of a radiation-absorbing tracer. The second class encompasses flow-type measurements which can be imagined by cutting a vessel, collecting a series of blood samples and measuring the tracer concentration therein. The concentrations ascertained using these approaches differ because particles with high flow velocity receive more weight in flow- compared to snapshot-type. The transport functions in the convolution chain in Eq. 2 belong to the flow type because a physical blood mixing at bifurcations is implied.

### Transport in a single vessel segment

Tracer inflow in the stem segment defines the initial and boundary conditions, which are necessary to predict the tracer flow in the entire arterial tree. Such conditions are straightforward to obtain in terms of an auxiliary function that describes propagation of an infinite bolus with a unit tracer concentration. This initial condition corresponds to labeling blood to the left from position *x* = 0 in Fig. 2 at the initial time moment *t* = 0. As demonstrated in the Methods section, the observed tracer concentration at a position *x* along the segment at time *t* after entering the segment takes the following form for the laminar flow and the snapshot-type measurements:
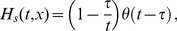
(4)where 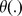 is the step function, which is zero for negative and unity for positive arguments and 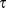 is the arrival time of the first tracer particles, 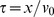, where 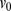 is the flow velocity in the fastest central streamline. For the flow type measurements



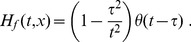
(5)
Equation 4 and Eq. 5 are obtained for idealized boundary and initial conditions described above. Realistic blood labeling can be taken into account using the linearity of the tracer propagation by adding to the above results the propagation of fictitious auxiliary volumes with initial concentration 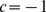 in the regions with no labeling. For example, for obtaining the propagation of an initial labeling at the location 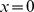 during a time interval 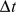, it is sufficient to subtract from Eq. 4 and Eq. 5 the same functions with the substitution 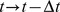. An explicit example of such calculation for our experiment is given in Methods section.

The tracer concentration for arbitrary rather than rectangular labeling is similarly obtained by first considering temporal labeling when a vessel originates from a well-mixed reservoir with a time-dependent tracer concentration, 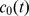. Taking infinitesimal 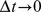 in the above example and using the linearity of tracer concentration, we obtain
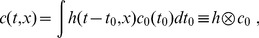
(6)where



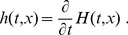
(7)This is applicable to both 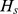 and 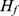. The transport function *h* takes the following explicit form for the radially parabolic Poiseuille flow:
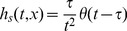
(8)

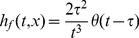
(9)The tracer can also be produced by instantaneous labeling with arbitrary spatially variable labeling intensity 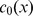 along a vessel segment. This gives the concentration
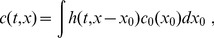
(10)where
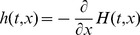
(11)for both 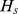 and 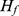. Note that this *h*-function is the distribution of distances travelled in time *t* rather than the travel time distribution.

### Illustrative experiment

We present here an experiment in which blood transport was monitored in large brain arteries in a healthy volunteer using magnetic resonance imaging. The technique of arterial spin labeling (ASL) is described in Methods section. Such an observation falls outside the scaling approach described above, but it illustrates the present theory in application to vessel segments with known morphology. The results further illustrate the ill-posed nature of the problem of model selection using indirect measurements such as those typically available with in vivo imaging.

**Figure 3:**
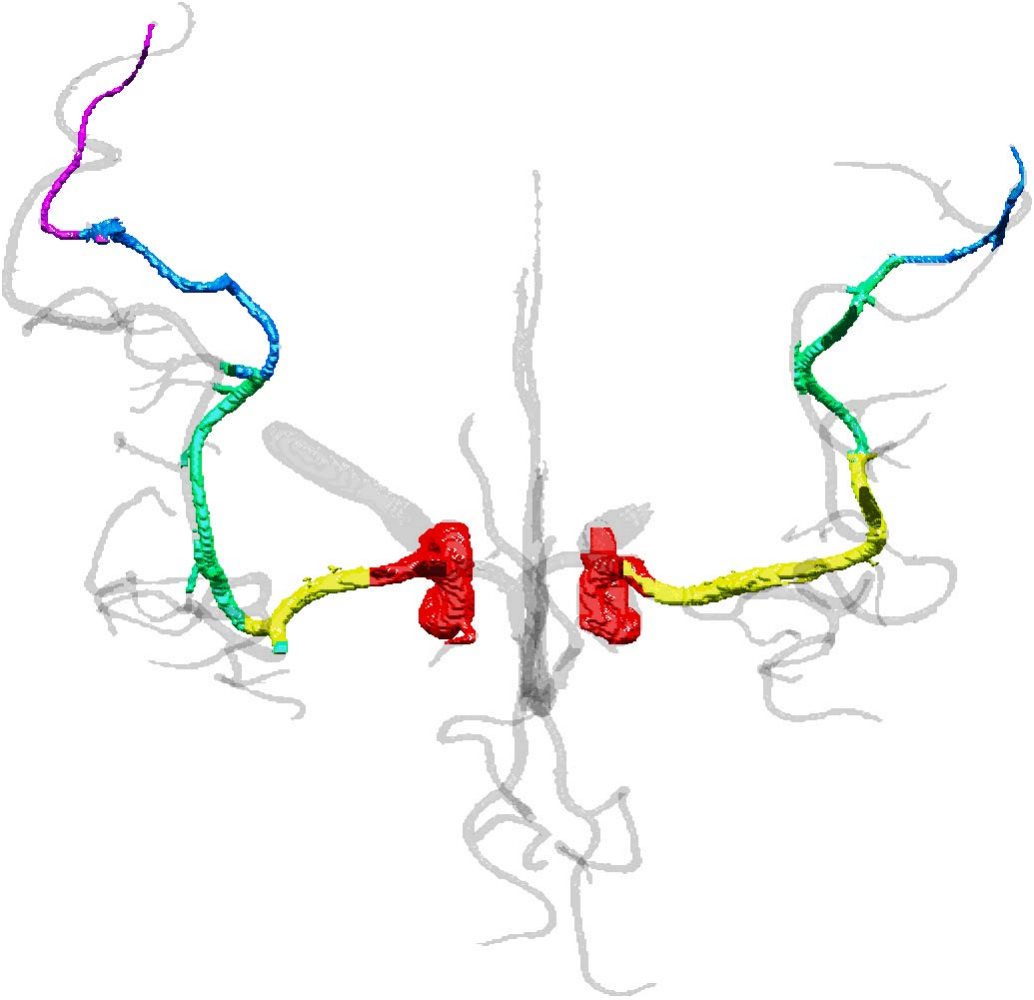
Internal carotid and middle cerebral arteries imaged with MRI. In both hemispheres a track with symmetric bifurcations was selected. Segments between bifurcations are shown with different colors. Model of tracer transport was specified for the left and right arteries up to unknown velocities in the stem segments (colored in red) using Eq. 2. The stem velocities were found by fitting the model to measured time-resolved passage of labeled blood in the arterial paths shown in colors.

We monitored arterial blood that was labeled in the cervical arteries 1cm below the skull base. We selected two paths beginning at the C1 parts of the internal carotid arteries and going downstream of the left and right middle cerebral arteries with nearly symmetric bifurcations, Fig. 3.

According to the above principles, the form of the transport function was predicted along each arterial path in terms of a single parameter that was the maximum flow velocity in the corresponding stem segment. We used morphological information available from MRI instead of scaling approach. The so obtained model included one more additional scaling parameter for the image brightness in each volume element (voxel) of three-dimensional image.

For the sake of comparison, we also present results obtained using two alternative mathematical models of the bolus shape. In the first, the total transport function 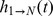 was obtained by replacing all transport functions in the convolution chain in Eq. 2 with the snapshot-type functions, 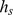. In the second model, theoretical prediction for observations was set to Gamma-variate curve
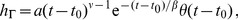
(12)which is widely used to describe any kind of intravascular bolus passage because this function has a reasonable shape including the exact zero before the arrival time. We fitted this model to our data to obtain benchmark for the fitting quality since this extremely flexible model has four adjustable parameters per voxel including the normalization factor, *a*, as explained in Methods.

**Figure 4:**
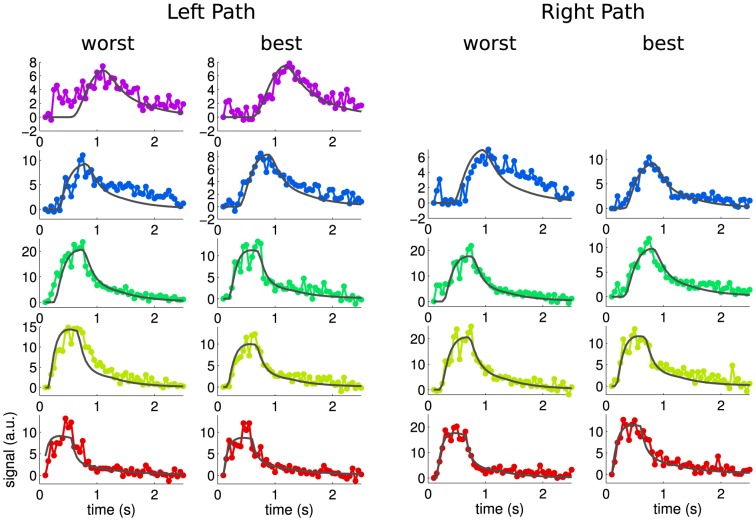
Fitting results for both arterial paths shown in Fig. 3. For each segment (colors from Fig. 3) worst (left columns) and best (right columns) fits are shown.

Figure 4 shows fitting results in different voxels selected as the worst and the best ones according to the 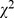 values for each generation of both paths. The difference between the best and the worst results is moderate while the overall fitting quality is good given the visible noise level.

**Figure 5:**
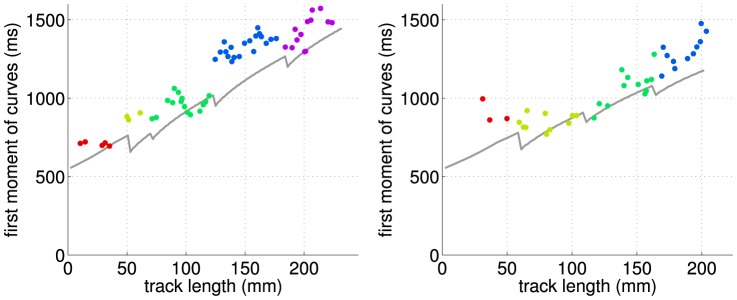
First moment of ASL bolus vs arterial track length for the left and right hemisphere (colors from Fig. 3). Note that the prediction from theory (solid line) is not a fit in this representation but was determined from the optimal *v*_0_ for the proposed 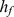 model (Table 1). The mixing at bifurcations is reflected in the sawtooth structure of the line.

Another test of our model is presented in Fig. 5 in which the first moment of the bolus time course, 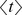 is shown as a function of the path length along the arterial for all voxels. The first moment of the ASL bolus time course is selected as a joint measure of delay and dispersion. In this case, the delay equals the bolus arrival time, 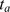, and the dispersion is characterized by the bolus first moment calculated when the origin of the time axis is set to the arrival moment, 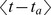. The first moment equals the sum of the so defined delay and dispersion.

The theoretical model in this figure is given by Eq. 2 with an additional convolution representing the transport from the last bifurcation to the measurement site:
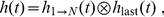
(13)where 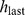 is of the *s*-type, Eq. 8, in our experiment. When marching along the arterial path and passing a bifurcation, the snapshot-type 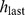 is replaced with a flow-type function for the last segment (due to physical blood mixing at the bifurcation), becoming part of 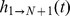 and additional 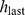 is added to describe the new segment just after the bifurcation. This results in a sawtooth-like structure. However, the present experimental data cannot provide support for this fine effect owing to a relatively high noise level.

Quantitative results are collected in Table 1. Fit of the proposed model yields maximum velocities 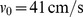 in the left and 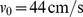 in the C1 segment of the right and left internal carotid arteries, respectively. This is in a good agreement with the reported cardiac-cycle averaged peak flow velocity of 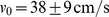 measured with Doppler ultrasound [11]. Several groups report the systolic peak flow velocities in the internal carotid artery which are 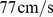 [12], 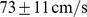 in segment C1 [13] and 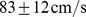 in segment C5 [14]. Analysis of flow obtained in a large-scale model of carotid bifurcation [15] suggests that the cardiac cycle averaged flow is about 50% of the systolic one. With this correction in mind, our results agree well with all cited data.

**Table 1:** Fitted maximal velocity, 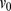, in the internal carotid artery and 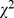 for different models (arbitrary units on common scale).

Model		left	right
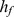	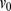	41 cm/s	44 cm/s
	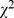	300	280
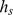	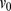	52 cm/s	45 cm/s
	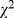	230	270
Gamma-variate	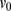	n.a.	n.a.
	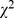	170	252

The Gamma-variate model does not include 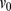 as a parameter.

**Figure 6:**
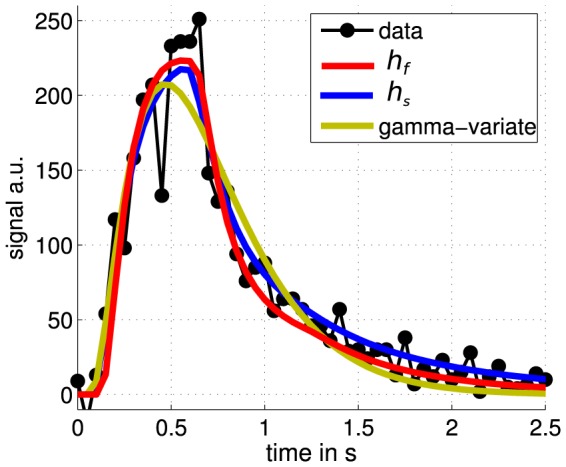
Comparison of different models for one voxel.

Results of fitting alternative models are also shown in Table 1. The pure snapshot-type model as described above by using exclusively 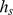, Eq. 8, instead of 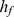, Eq. 9, results in a slightly better fitting accuracy and a flow velocity in the same physiologically reasonable range. The Gamma-variate model performed similarly in spite of a much larger number of voxel-related adjustable parameters (see also the exemplary model comparison in Fig. 6).

### Correction of arterial input functions

Perfusion evaluation with many imaging modalities typically has to rely on arterial input functions (AIF) measured distant to their theoretically required position. In PET for example, the arterial tracer concentration can be measured with high precision using blood sampling. However, the shape of the estimated AIF is significantly broadened due to transport in the catheter of the measurement device. The proposed model can straightforwardly be applied to that system.

In perfusion MRI, it is known that determining the AIF at a location remote to the tissue leads to underestimation of cerebral blood flow due to vascular transport effects [16]. We applied the present theory to measurements of cerebral blood flow with the AIF determination in the carotid arteries. As explained in Methods, this indicates an underestimation of the blood flow by approximately 20%, which is in line with the MRI – PET comparison in the porcine model [17].

## Discussion

### Summary of the Model

We present here a model of tracer dilution during its transport through the arterial tree. For the conventional injection of tracer, the model is formulated in the framework of a transit time distribution. The kernel distribution function is found explicitly for the laminar flow in cylindrical vessel segments.

We draw attention to the dependence of the measured tracer concentration on the measurement type and discuss two of them: (i) a snapshot type when the tracer concentration is measured instantly in a given volume and (ii) a flow-type when the tracer concentration is evaluated at a given location during a time interval. The snapshot-type refers to the apparent mean concentration in a vessel lumen containing simultaneously different local concentrations whereas the flow-type describes a true mixing of the fluid at a given location.

As a general note, the common interpretation of 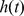 in Eq. 1 as the transit time distribution does not specify this function unambiguously. The remaining freedom is selection of population of tracer particles contributing to the distribution. The two measurement type discussed in this study realize two possible choices of such a population.

Vessel bifurcations essentially complicate the overall transport process. We show that the approximation of a good mixing at bifurcations simplifies the problem enormously so that the overall transport is reduced to a combination of the transport functions of individual segments via a convolution chain, Eq. 2. The good mixing implies that upon averaging over many typical bifurcations, the blood particle velocities before and after the bifurcation are statistically independent. The combination of this model with a scaling model of the arterial tree results in an extremely simple description of transport in such a tree.

Representation of the transport kernel in the form of a convolution chain, Eq. 2, suggests convenient temporal characteristics of the tracer bolus selected by their mathematical properties. First, the arrival time 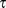 is additive in the convolution being equal to the sum of arrival times in each individual segment. So is the mean transit time, 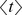, which is the first moment of the distribution 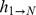. The difference 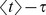 characterizes the bolus dispersion and is obviously additive.

Note that the distribution of transit times for a parabolic velocity profile has a long tail, which decreases as 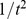 for 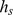 or 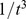 for 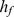 due to the low fluid velocity near the vessel wall. This results in the non-existence of the mean transit time for the snapshot-type measurement which is the first moment of this distribution. In practice, this does not create a problem for the conventional tracer kinetics, Eq. 1, including the central volume principle because this framework implies the flow-type measurements.

The snapshot-type transport was confirmed with a high accuracy in our previously reported experiment [18] in which the tracer concentration was measured using bolus-tracking MRI in a hose that was attached to a mixing reservoir with a known concentration time course.

### Limitations and perspectives

The assumption of good blood mixing at vessel bifurcations is central for the presented description of the entire vascular network. It should be understood in the statistical sense as the absence of correlations in the blood particle velocity before and after the bifurcation after averaging over the entire vasculature. This is feasible in view of the variability of the precise vessel shape at bifurcations, but a proper verification involving computational fluid dynamics remains a subject of future research.

Good mixing only applies for bifurcations with nearly equal calibers of daughter vessels. Such bifurcations are relevant for the terminal distribution rather than long-distance delivery part of the tree when the vessel caliber is related to the length of its crown formed by all daughter vessels [19]. In contrast, small offsprings of larger long-distance-delivery arteries receive the slowest blood flowing close to the vessel wall. A possible way to take such bifurcations into account is to introduce an effective reduction in blood flow along vessels involved in the long-distance blood transport.

The statistical distribution of blood flow in small arteries is shown to approach log-normal due to cumulative effect of asymmetric flow splitting at bifurcations [20]. This derivation ignored other sources of dispersion such as one within vessel segments which is the focus of the present study. Incorporation of the uneven flow distribution at bifurcations in the spirit of Ref. [20] is a straightforward future extension of the present work.

Closed-form expressions for the transport functions are obtained for the steady laminar flow in cylindrical vessel segments. This assumption is violated at both ends of the arterial tree. First, blood flow in the largest stem arteries is pulsatile. The effect of pulsations on cerebral blood flow measurements has been discussed in [21] and [8]. Gallichan et al. [8] concluded that averaging over multiple heart cycles without cardiac gating closely matches the concentration curves obtained with steady flow.

Second, flow is not laminar in the smallest arteries due to the presence of red blood cells as is well known in rheology [22], [23]. Erythrocytes disturb plasma flow resulting in an effective mixing. These effects can be neglected in vessels with large radii, 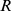, such that 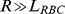, where 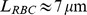 is the size of erythrocytes. Further, the tracer kinetics is affected by diffusion in the direction transverse to the flow [24]. This effect is maximal when water itself serves as tracer for which case we perform the following estimate. Water molecules diffuse over a typical distance 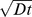, where 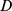 is the diffusion coefficient and 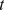 is the time available for diffusion. The derived transport function can be considered valid as long as this distance is much shorter than the vessel lumen. Using 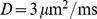 and 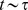, where 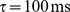 is the transit time through a single vessel segment we obtain a limitation for the vessel radius 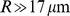. Note that below this limit, when lateral motion effects are dominant, the difference between the snapshot-type and the flow-type measurement disappears.

Summarizing these limitations, the proposed model is applicable to the major middle part of the arterial tree between the largest cerebral arteries and the smallest arterioles. In particular one can apply it to describe blood transport from large arteries, which are still resolvable in MRI, PET or CT images to those with the length comparable to the voxel size of about a millimeter.

The model for the vasculature derived from the scaling rules is certainly an oversimplified perspective onto the human brain vasculature. A more realistic description is available for smallest vessels [19], [25]–[28] and for broader range of vessel diameter [29]. Deviations from Murray's law have been addressed in several studies [19], [27], [30], [31]. Some diversity in reported results should be understood in view of different species and organs investigated. The present framework can incorporate scaling models of microvasculature by systematic parameter change along the convolution chain in Eq. 2 and Eq. 13. A more challenging problem is to extend the present model to small vessels with account for realistic rheology and transverse diffusion.

### A challenge of accessing vascular morphology using tracer kinetics

In spite of the limited applicability to large vessels, our model fits well the data obtained using arterial spin labeling. With its single adjustable parameter per voxel and one global per artery, the model yields reasonable cerebral blood flow velocities in the middle cerebral artery when the distal part of the internal carotid artery is used as stem vessel [11]–[14].

This model described the data only slightly worse than the very flexible Gamma-variate function with its four parameters per voxel. This suggests that the fitting accuracy is limited by the noise present in data: More flexibility in the fitted function does not result in a substantial reduction in 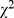 as long as overfitting does not take place. Beyond the smaller number of parameters, an advantage of the present model is a clear biophysical meaning of these parameters, while the parameters of the Gamma-variate function are not straightforwardly interpretable.

On the other hand, the good fitting quality of the proposed model cannot be automatically inverted to a conclusion about its validity. This is demonstrated by replacing the main building block of our model, 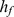, with 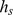, Eq. 8 and Eq. 9, which resulted in a slight decrease in 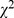. A good fitting quality of such 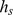 model without account for vessel bifurcations has been reported also in Ref. [8].

Such “fitting tolerance” can be understood in view of the fact that the expected bolus dispersions using 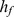 and 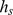 differ only slightly after passing through few bifurcations, but the difference accumulates for large arterial trees all the way to the capillaries. This suggest that a proper validation may be performed by measuring blood flow in a larger number of smaller vessels, that is for higher generations within the arterial tree. Such a verification still remains a challenge, although the results presented here encourage to face it. The outcome may be a new approach to in vivo examination of statistical microvascular morphology.

### Correction of arterial input functions

The present model predicts an underestimation of cerebral blood flow of about 20% due to measuring an arterial input function at a location distant to the tissue of interest. This agrees well with our recent comparison between MRI and PET in the porcine model [17] and other studies on cerebral [32], [33] and myocardial perfusion [34]. A correction for this effect appears feasible in normal tissue, but problematic in thrombotic or embolic stroke in which case the disturbance of blood flow depends on the location of the arterial occlusion, see e.g. Ref. [35] for a discussion.

## Conclusions

Two findings are reported here. First, we have shown that experimentally measured tracer concentration in a vessel segment depends on the measurement type and we derived an explicit new form of the transport function for the case of blood collected at an end of a straight cylindrical vessel. Second, we have shown that the approximation of good blood mixing at vessel bifurcations results in a drastically simplified description of the entire arterial tree with explicit results for the case of its self-similar structure. Arterial spin labeling measurements in large brain arteries support the developed model although are not sufficient for an unambiguous verification. Application of this model to cerebral blood flow measurements with a distal arterial input function suggests a 20% underestimation of blood flow, which is in agreement with a recent comparison of cerebral perfusion evaluations with MRI and PET.

## Methods

### Transport in a single vessel segment

**Figure 7:**
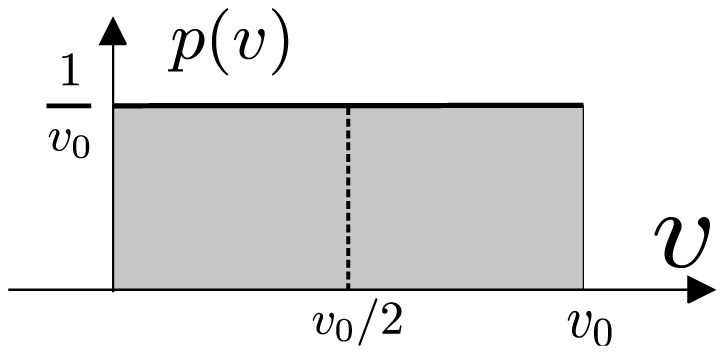
Laminar flow in a cylinder forms a radially parabolic velocity profile for which the distribution of velocities is uniform in the interval 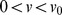
 **.** The mean flow velocity is 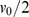.

We present here a derivation of Eq. 4–Eq. 11. The transport function of a segment between bifurcations is determined by the distribution of fluid velocities in the vessel cross-section, 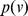. This distribution is uniform for Poiseuille flow 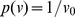 for 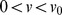, where 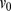 is the maximal flow velocity in the central streamline, and zero otherwise (Fig. 7).

Snapshot-type measurements take place when the concentration is measured at time 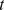 in a small volume at position 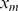 as in Fig. 2 a). The fluid is not really mixed in this case and the concentration of the tracer must be understood in the average sense: The amount of tracer inside the volume element divided by its volume. That is, the average tracer concentration 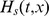 is determined by the volume fraction of the central part of the paraboloid in Fig. 2 a), in which the tracer with initial concentration 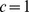 has already arrived:
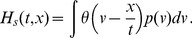
(14)The step function 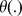 under the integral ensures that only particles with velocities greater than 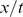 contribute to the concentration. The measurement position 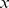 enters the result via the ratio 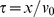. Substitution of 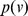 with the uniform distribution results in Eq. 4.

Flow-type measurements take place when the fluid is physically mixed (e.g at a bifurcation). The tracer concentration is given by the ratio of the amounts of tracer and blood that have crossed a selected vessel cross-section by the time 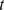 as in Fig. 2 b). The resulting tracer concentration, 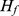,
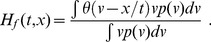
(15)Substitution of 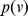 with the uniform distribution results in Eq. 5.

Consider a vessel originating from a well-mixed reservoir with a time-dependent tracer concentration, 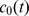. First, let 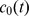 be a rectangular pulse with a unit height and a duration from 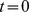 to 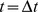. The trailing edge of the label is introduced by propagating fictitious volume with label concentration set to 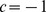 at the labeling site after 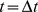:
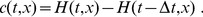
(16)Taking the limit 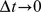 and summing over all time moments weighted with the corresponding 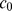 results in Eq. 6 and Eq. 7.

The labeling can also be performed spatially by instant labeling of blood. Consider first labeling applied on an interval 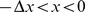. The absence of labeling at 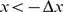 is described by propagating a fictitious volume with label concentration set to 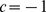 for all 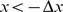. This gives the concentration
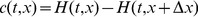
(17)for both 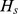 and 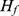. Locating initial labeling near a point 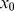 instead of 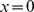 results in replacing 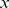 with 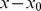 in the above expression. The tracer concentration for an arbitrary initial profile 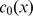 results now from considering infinitesimally small 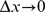, and summing the resulting concentrations for different locations, 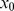, of so obtained thin slices weighted with the initial spatial concentration profile. This results in Eq. 10 and Eq. 11.

Note that injection of a contrast agent, as well as the mixing process in a reservoir correspond to a temporal labeling. With ASL, the tracer is blood itself, in which magnetization of water protons is manipulated using radio frequency pulses. With this technique, the labeling can be performed both temporally (continuous ASL) and spatially (pulsed ASL). In this context, the explicit form of Eq. 17 has previously been reported for the case of the snapshot-type measurement [8].

### ASL Measurement and data processing

We performed a measurement in a healthy volunteer in accordance with the Helsinki Declaration of 1975. The study was approved by the local ethical board of Fraunhofer Institute for Medical Image Computing MEVIS and the volunteer gave written consent. Magnetic resonance was used to label arterial blood in the cervical arteries and to image it in the brain volume with 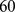 time steps starting at 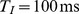 with an increment of 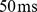 with a nominal spatial resolution 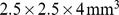. Morphological information was obtained using MRI time-of-flight angiography (3D-TOF-MRA) with a nominal spatial resolution 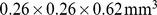.

Modeling the blood flow begins with a description of the excited ASL bolus. In our implementation, excitation of the initial slab (crf. Eq. 17) is followed by a cut-off pulse after 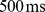 (crf. Eq. 16). The ASL bolus is described by linear combinations of functions given in Eq. 17 and Eq. 16 according to the initial and boundary conditions (explicit analytical description is given below). The present measurement of blood magnetization belongs to the snapshot type, which implies the use of 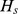 functions, Eq. 4. After the first bifurcation, the functions 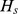, are replaced with 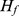, Eq. 5 to account for the mixing at the bifurcation. The transport function in the second vessel segment is obtained via a convolution with 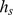 according to Eq. 13. The factor 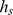 turns in 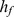, Eq. 2 immediately after the next bifurcation. Continuing this procedure, we build a model for the selected part of the arterial tree.

**Figure 8:**
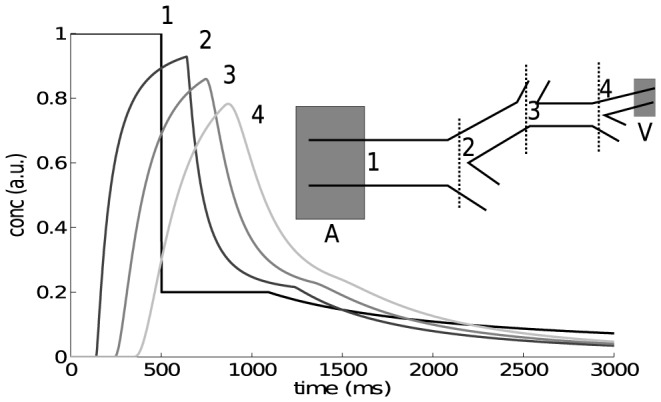
ASL Model (without T_1_ relaxtion correction): Concentration time curves at 4 different positions in the vascular tree. The evolution of the ASL bolus from the labeling region A to a measurement volume V is given with the convolution chain 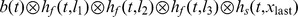, where 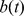 is the ASL bolus (1), the 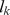 are the vessel segment lengths and 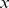 is the distance from the bifurcation in the last segment (4). The shape of 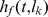 (2,3) is actually defined by the time constants 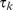.

To reduce the number of model parameters, we relate the flow velocity in the daughter segments at each bifurcation to the velocity in the parent segment using the flow conservation, the measured segment cross-sectional areas and assuming the Poiseuille flow with equal flow splitting at symmetric bifurcations. Hence, the flow velocities in all segments are recursively determined by the velocity in the stem segment. With the known segment dimensions, the flow velocities are related to the time constants 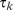 that actually define the shape of the transport functions in Eq. 2. Thus the maximal flow velocity in the stem vessel is the only parameter to be determined from experimental data. Finally, relaxation of the water protons spins is accounted for by a factor 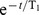 with 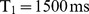, the longitudinal relaxation constant of blood at 3T [36]–[38]. The resulted transport functions are shown in Fig. 8.

The maximum velocity in each stem artery was determined by minimizing 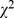:
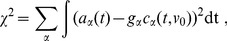
(18)where 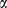 counts voxels within the selected arterial path, 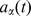 is the measured signal time course, 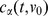 is the theoretical curve and 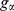 is a scaling parameter. The latter is in principle global for the whole data set reflecting its arbitrary brightness scale, but the presence of partial volume effect, some cross-talk between image voxels and other imaging artifacts suggest treating it as a local parameter. Minimization with respect to 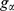 was performed analytically. Our model, thus, includes 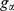 as a single parameter per voxel in addition to one global parameter 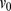 per arterial path.

#### Model for ASL Measurement

**Figure 9:**
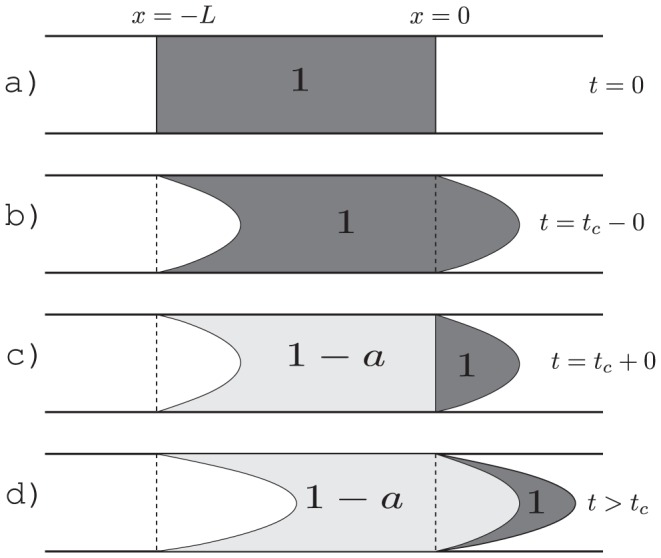
Formation of the ASL bolus: The bolus is initially created with a defined spatial length (a) and evolving (b). After time 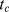, an additional saturation is applied to create a bolus with a defined temporal duration, where only previously inverted magnetization is affected. As the saturation efficiency is only 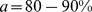, there is still a contribution from the initial excitation, now with the weight 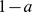 (c). Thus, the labeling process creates two different compartments which are evolving further (d).

We describe here a model for our ASL experiment. The evolution of a pulsed arterial spin labeling (PASL) bolus can be described by Eq. 17 in the main text. However, the bolus excitation included an additional saturation pulse applied to the labeling region in order to define a certain bolus duration. The saturation reduces the magnetization of already labeled blood with an efficiency of 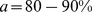, but does not affect non-labeled blood. The most straightforward way to describe the initial bolus is to decompose the necessary initial conditions in a sum of instant labeling in a half-infinite interval to different time moments. This gives the corresponding combination of 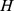-functions for the ASL bolus, 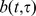 as illustrated in Fig. 9:
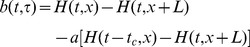
(19)where 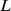 is the length of the initially excited bolus and 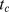 is the bolus duration. This equation is applicable to both the snapshot and the flow-type measurements.

For the snapshot type measurement Eq. 19 takes the explicit form
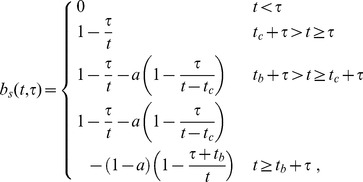
(20)where 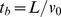. This expression has been first obtained in [8] for the case 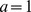. As discussed in the main text, the function 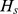, Eq. 4, in Eq. 19 and Eq. 20 are to be replaced with 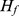, Eq. 5 after the first bifurcation. Explicitly, this implies replacing 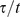 in Eq. 20 with 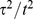 and making analogous substitutions for other entries of time.

The recursive determination of the transport functions in successive segments relies on the expression 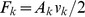 for the flow in the 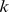
'th segment with a parabolic velocity profile. Here 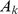 is the segment cross-sectional area and 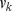 is the central streamline velocity. The travel time in this streamline is the time constant 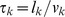, where 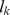 is the segment length. The conservation of flow relates the 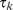 of each child segment to its parent: 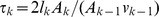 for symmetric bifurcations. The only undetermined parameter is the time constant 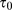 (or the corresponding velocity 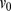) in the stem segment.

#### Data processing

All data processing was performed using MATLAB (Mathworks, Natick, USA). To segment the vascular tree, the TOF images were thresholded and a flood-fill algorithm was applied with the internal carotid artery as a seed point. There are numerous strategies for automatic tracking of vessels [39]. However, because we want to test the model under controllable conditions, the bifurcation detection and vessel tracking was performed semi-automatically. First, symmetric bifurcations were identified downstream of internal carotid arteries in each hemisphere. The path between the selected bifurcations was then found automatically by following the center of mass of the segments. The track length and the volume of each segment were calculated by counting the number of contained voxels. The cross sectional area was computed by dividing this volume by the length of the segment. With this morphological information 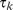 for each segment was calculated as described above. The voxels of the segmentation were mapped to the ASL measurement with coarser resolution and the measured time series of the ASL signals were used for further data processing.

### Evaluation of transport effect on arterial input functions

Assume that an arterial input function is measured in common carotid arteries for evaluation of cerebral perfusion. The effect of such a distal measurement is a replacement of the tissue residue function, 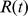, with a convolution with a transport function, 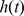, describing the blood transport from the measurement site to the tissue, 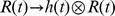. According to theory, the quantity defined from measurements is the product 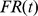, where 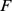 is the blood flow. Using the theoretical property 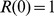, one finds 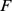. In practice, the residue function is smoothed and one uses its maximum as an estimate for the magnitude at the initial time moment. Our numerical simulations indicate an about 20% reduction in the height of 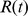 due to the transport effect, which becomes the underestimate of blood flow in the standard data processing.

The effect magnitude can be understood in simple terms using the following estimate. First, note that the convolution with the transport function does not change the time integral of 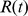; some broadening of the function's shape is compensated by a reduction in its height. The observed blood flow is thus inversely proportional to the width of 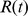. The latter can be estimated as the first moment 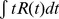, which equals the second moment of the underlying transit time distribution within the capillary bed of investigated tissue, 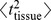. Applying this to the genuine 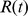 and to 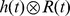 and using the fact that first moments are additive in convolutions, we obtain
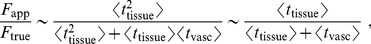
(21)where 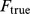 and 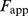 are the genuine and the apparent blood flow, respectively, 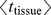 is the width of 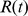 (the tissue mean transit time) and 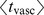 is the width of 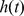 defined as its first moment. The replacement of the second moment of transit time in tissue with its first moment squared is exact for exponential residue functions. For other shapes, it is understood as an order-of-magnitude estimate.

We do not possess data for these times, but they can be estimated using the scaling model of the arterial system. We assume a reduction in the arterial diameter from 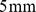 to 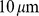 for the carotid arteries and the smallest arterioles, respectively. This results in 27 vessel generations when each generation gets the factor 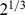 smaller than the previous one according to the Murray's law. So does the vessel segment length. The true arterial input can be defined as the tracer concentration in arteries that are as long as the imaging voxel, for example 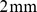 for MRI perfusion measurements. Assuming the length 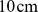 for the largest cerebral arteries in which the arterial input is measurable, we obtain 17 generations between them and the true arterial input.

For an estimate of the time constants of the segments we need the flow velocity. An estimate is obtainable from our results for the selected arterial paths although the scaling is hardly applicable to the large arteries. The fitting results shown in Fig. 4 give 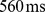 for 5 generations and 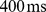 for 4 generations for the left and right paths, respectively. The mean is 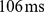 per generation. This gives 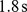 and 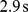 for the arrival time to the location of the true arterial input and to the vascular bed, respectively. Note that although very approximate, this estimate gives a reasonable blood velocity in the capillaries, which can be estimated using the velocity of red blood cells 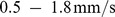 in rats [40] and 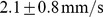 in cats [41]. Using 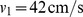 as the mean for both arterial paths, we obtain 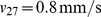 after 27 vessel generations.

Using the above estimates, we obtain 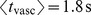 for 17 generations. This is 23% of the average mean transit time of about 8 sec, which we recently measured in the porcine model [17]. This indicates an underestimation of the blood flow by approximately 20%, which is in line with the MRI – PET comparison of the same study. In humans, reported values for the mean transit time are generally lower, between 3 and 6 s [42]–[45]. In this case, the underestimation is greater, accounting for about 35% of the true flow. This is in the same range as the values usually reported in other simulation studies [46], [47].
